# Neurofibromatosis type 1 is not associated with subarachnoid haemorrhage

**DOI:** 10.1371/journal.pone.0178711

**Published:** 2017-06-02

**Authors:** Arttu Kurtelius, Roope A. Kallionpää, Jukka Huttunen, Terhi J. Huttunen, Katariina Helin, Timo Koivisto, Juhana Frösen, Mikael von und zu Fraunberg, Sirkku Peltonen, Juha Peltonen, Juha E. Jääskeläinen, Antti E. Lindgren

**Affiliations:** 1 Neurosurgery, NeuroCenter, Kuopio University Hospital, Kuopio, Finland; 2 Neurosurgery, Institute of Clinical Medicine, University of Eastern Finland, Kuopio, Finland; 3 Department of Cell Biology and Anatomy, Institute of Biomedicine, University of Turku, Turku, Finland; 4 Department of Dermatology, University of Turku and Turku University Hospital, Turku, Finland; Universitatsklinikum Freiburg, GERMANY

## Abstract

**Background:**

The prevalence of intracranial aneurysms (IAs) has been proposed to be elevated in the patients with neurofibromatosis type 1 (NF1). Our aims were to determine the prevalence of NF1 in a large Finnish population based cohort of IA patients and, on the other hand, the occurrences of subarachnoid haemorrhage and unruptured intracranial aneurysms in a nationwide population-based cohort of NF1 patients and its matched ten-fold control cohort.

**Methods:**

The Kuopio IA Database (www.kuopioneurosurgery.fi) includes all ruptured and unruptured IA cases admitted to the Kuopio University Hospital (KUH) from its defined Eastern Finnish catchment population since 1980. In this registry-based study, we cross-linked the Kuopio IA database with the Finnish national registry covering all hospital diagnoses. The NF1 diagnoses of the 4543 patients with either saccular of fusiform IA were identified from 1969 to 2015 and verified from patient records. Our second approach was to analyze the occurrence of aneurysmal subarachnoid haemorrhage (aSAH) and unruptured IAs in a nationwide population-based database of 1410 NF1 patients and its ten-fold matched control cohort (n = 14030) using national registry of hospital diagnoses between 1987 and 2014.

**Results:**

One NF1 patient was identified among the 4543 IA patients. Three verified IA cases (one unruptured IA and two aSAH cases) were identified in the cohort of 1410 NF1 patients, with similar occurrences in the control cohort.

**Conclusions:**

We found no evidence in our population-based cohorts to support the conception that NF1 is associated with IAs. Our results indicate that the incidence of aSAH is not elevated in patients with NF1. Further studies are required to confirm that there is no association between NF1 and unruptured IAs.

## Introduction

Neurofibromatosis type 1 (NF1) is a relatively common autosomal dominant genetic disorder caused by a mutation in the *NF1* gene on the long arm of chromosome 17.[1] The clinical hallmarks of NF1 include café-au-lait spots, neurofibromas, and Lisch nodules of the iris, yet NF1 can affect several organ systems (Tables 1 and 2).[2] Even though NF1 displays 100% penetrance, the clinical severity is highly variable even within one NF1 family.[3] Cardiovascular complications that have been associated with NF1 include stenosis of pulmonary, renal, mesenteric and intracranial arteries, moyamoya disease–and intracranial aneurysms (IAs).[4–8] The association between IAs and NF1, however, has been contentious during the last two decades.[6,9–11]

**Table 1:** Diagnostic criteria for NF1: Two or more of the following findings[2].

Six or more café-au-lait macules with diameters >5 mm in prepubertal and >15 mm in postpubertal patients
Two or more neurofibromas of any type or one plexiform neurofibroma
Axillary or inguinal freckling
Optic glioma
Two or more Lisch nodules of the iris
A distinctive osseous dysplasia (sphenoid wing dysplasia, bowing of the long bones, or pseudarthrosis)
A first degree relative diagnosed with NF1

**Table 2:** Manifestations of NF1 in different organ systems[8].

Pigmentary abnormalities	Café-au-lait macules, axillary freckling, Lisch nodules
Neurofibromas	Benign Schwann-cell tumors containing several cell types
Plexiform neurofibromas	Benign tumors arising from multiple nerve fascicles and growing along nerves. Usually congenital but have potential to grow and become malign
Skeletal deformities	Osteoporosis/osteopenia, dystrophic scoliosis, sphenoid wing dysplasia, tibial dysplasia and pseudarthrosis, short stature, increased head circumference
Cardiovascular abnormalities	Idiopathic and secondary hypertension, vascular dysplasia
Neurocognitive deficits	Visuospatial and visuomotor deficits, learning disabilities, ADHD
Tumours of nervous system	Glioma of the optic pathway, malignant peripheral nerve sheath tumors, brainstem glioma, glioblastoma
Other tumours	Gastrointestinal stromal tumors, breast cancer, leukaemia and lymphoma, especially myeloid leukaemia, phaeochromocytoma, duodenal carcinoids, rhabdomyosarcomas.
Endocrine abnormalities	Precocious or delayed puberty
Other manifestations	Focal T2 hyperintensities of the brain (UBOs)

IAs are estimated to be carried by approximately 3% of the general population.[12] Known risk factors for IAs include age, female sex, smoking, hypertension, family history for IAs and autosomal dominant polycystic kidney disease (ADPKD).[12,13] The genetic mechanisms predisposing to the complex IA disease are, so far, unclear.[14]

We cross-linked two large Finnish population-based patient cohorts with the hospital diagnoses from the nationwide registry. Our aim was to find out if there is any additional evidence to support the consumption that NF1 is associated with intracranial aneurysms by determining the prevalence of NF1 in a population-based cohort of IA patients and, on the other hand, the prevalence of SAH and IAs in a national register of NF1 patients.

## Methods

The Kuopio IA Database (www.kuopioneurosurgery.fi) includes all ruptured and unruptured IA cases admitted to the Kuopio University Hospital (KUH) from its defined Eastern Finnish catchment population since 1980.[15] The prevalence of NF1 in the 4543 patients with unruptured IA or aSAH, as well as their 19644 first-degree relatives, was analyzed. In a reverse approach, the 1410 NF1 patients of the Finnish NF1 Registry were analyzed for unruptured IA and aSAH diagnoses between 1987 and 2014.[16]

### KUH catchment population

Neurosurgery of Kuopio University Hospital (KUH) has been the sole provider of full-time acute and elective neurosurgical services in its catchment area in Eastern Finland since 1977. The catchment area consists of four central hospitals, each with their own neurological units and catchment areas. During the recruitment period of the present study, from 1980 to the end of 2015, the geographic KUH area has not changed. The population has decreased from 882 671 to 815 021.^17^ The median age has risen from 37 to 42 years in men and from 40 to 45 years in women, while the proportion of men has remained at 49%.[17,18]

### Kuopio intracranial aneurysm patient and family database

All patients admitted to KUH with unruptured or ruptured IAs have been recorded in the Kuopio IA Database (www.kuopioneurosurgery.fi) since 1980, prospectively since 1990. The clinical data from the hospital periods and follow-up visits have been recorded. The family history for IA has also been recorded: an IA family contains at least 2 affected first-degree relatives.[18]

### Identification of NF1 in 4543 IA patients in Kuopio IA Database

Clinical data including prescribed drugs and causes of death have been imported from the national registries.[15,19] The first-degree relatives of the 4543 IA patients were retrieved from Population Register Centre of Finland, using the personal identity codes, and their clinical data were also retrieved from the nationwide registries. The Kuopio IA Patient and Family Database was cross-linked with the Finnish electronic hospital diagnosis registry (Care Register For Health Care HILMO, managed by the Finnish Institution for Health and Welfare) that covers all secondary and tertiary centres in Finland and includes all medical specialties. Patients with NF1-related diagnoses were searched with the ICD-8, ICD-9, and ICD-10 codes (when applicable) for NF1, as well as unspecified neurofibromatosis (743.4, 237.70 and 237.71, and Q85.0 and Q85.00, respectively), café-au-lait spots (709.09 and L81.3), benign neoplasms of brain, cranial nerves and peripheral nerves (225, 225, and D33 and 225, 229, and D36), nerve root and plexus disorders (357, 353 and G54), mononeuritis of the upper and the lower limb (357, 354 and G55, and 355 and G56), disorders of continuity of bone (733.8 and M84), and malignant neoplasms of peripheral nerves (192.4, 192, and C47). The records of the patients potentially carrying NF1 were carefully re-reviewed to confirm or exclude the diagnosis of NF1.

The hospital diagnoses of the 19644 first-degree relatives of the 4543 patients were searched for NF1 (743.4, 237.70 and Q85.0). Finally, the patient records of the IA patients with first-degree relative(s) with NF1 were screened for signs of NF1.

### Identification of IA among 1410 NF1 patients in Finnish NF1 Registry

The Finnish NF1 Registry is a nationwide registry of all the Finnish NF1 patients who had visited any of the five university hospitals or 15 central hospitals of Finland between 1987 and 2011, with NF1 in their medical records.[16] The NF1 diagnoses have been ascertained by the re-review of the medical records, and all cases fulfil the NIH diagnostic criteria for NF1 (Table 1). The present NF1 cohort comprises of 1410 patients, 48% males. A ten-fold control cohort (n = 14030), individually matched by the age, sex and place of residence at the time of NF1 cohort entry, was retrieved from the Population Register Centre of Finland. The 1410 NF1 patients had been followed up from the first hospital visit associated with NF1 between 1987 and 2011, and the 14030 controls had been followed up from the NF1 cohort entry date of their respective NF1 patients. The follow-up time ended at death, emigration, December 31, 2014, or the date of occurrence of an IA diagnosis (unruptured IA or aSAH). The 1410 NF1 patients and their 14030 controls were linked with the same Finnish electronic hospital diagnosis registry (Care Register for Health Care) that was used to identify NF1 diagnoses in the IA Patient and Family Database, and patients with diagnoses of subarachnoid haemorrhage (SAH) (ICD-9 431, ICD-10 I60.1-I60.9) and unruptured IAs (ICD-9 437.3, ICD-10 I67.1) were identified. Their diagnoses were verified from medical records. In addition, the death records of the 249 NF1 patients deceased before July 1, 2013, were reviewed to identify the diagnoses of aSAH and unruptured IAs.

### Statistical methods

Incidence of diagnoses in the NF1 cohort was analyzed using the Cox proportional hazards model with stratification by a factor that indexed matched sets of NF1 patients and controls.

### Ethical aspects

The Kuopio IA Patient and Family Database has been approved by the Ethical Committee of the KUH, the Finnish Ministry of Social Affairs and Health, and the National Institute for Health and Welfare and written informed consent was obtained from all patients. The NF1 database was approved by the Ethics Committee of the Southwest Finland Hospital District, the Finnish Ministry of Social Affairs and Health, and the National Institute for Health and Welfare waiving the need for patient consent, since patients were not contacted during data collection.

## Results

### Prevalence of NF1 in the Kuopio IA cohort

Among the 4543 IA patients, a total of 156 patients with diagnoses potentially related to NF1 were identified. Only two of the 156 IA patients had a recorded diagnosis of NF1, but the other proved to have tuberous sclerosis (Q85.1), mislabelled as NF1. Of the 156 IA patients, another five had symptoms suggestive of NF1: however, one had clinical features and histological findings indicative of schwannomatosis; two had a confirmed diagnosis of neurofibromatosis type 2 (NF2); one had features indicative of NF2; and one had a suspected neurofibroma that turned out to be a leiomyoma. Five first degree relatives to the 4543 IA patients had the diagnosis of NF1, but none of their three IA carrying relatives had clinical signs of NF1. The prevalence of NF1 was one per 4543 (22/100000) in the IA cohort. The single IA patient with NF1 was a 46-year-old woman with incidental bilateral middle cerebral artery bifurcation IAs while her cerebral vasculature was otherwise normal in angiography.

### Diagnoses of unruptured IAs or SAH in the NF1 cohort

The 1410 NF1 patients and their 14030 matched controls had been followed up for totals of 21220 and 229307 person-years with mean follow-up times of 15.0 and 16.3 years, respectively. Only one unruptured IA was found in autopsy in the NF1 cohort and no cases of unruptured IA were reported during the follow-up, whereas 19 unruptured IA cases had been found among the controls during follow-up. During follow-up, five of the 1410 NF1 patients and 34 of the 14030 controls had been diagnosed with SAH, a hazard ratio of 1.8 (95% CI 0.69 to 4.67, *P* = 0.234), but in a closer review of patient records and death certificates, only one NF1 patient had had SAH of aneurysmal origin. In addition, one patient with aneurysmal SAH was found by reviewing the death certificates of the NF1 cohort.

## Discussion

### Key results

To our knowledge, this is the first comprehensive study on the occurrence of NF1 in a large population-based IA cohort (n = 4543), and unruptured IA or aSAH in a population-based NF1 cohort (n = 1410). Only one patient with confirmed NF1 was found among the 4543 IA patients admitted between 1980 and 2015 to KUH from its defined Eastern Finnish catchment population. On the other hand, only three patients with confirmed unruptured or ruptured IAs were found in the nationwide cohort of 1410 NF1 patients. In our cohorts we found no evidence that NF1 disease is associated with IA disease, suggested by previous studies (Tables 3 and 4) and literature reviews.[20]

**Table 3:** NF1 cohorts with intracranial aneurysms.

Author	Country	Type of study	No. of NF1 patients (No. of patients with imaging)	Mean age ± SD	Imaging and diagnosis of cerebrovascular abnormalities	No. of IA patients	No. of patients with other cerebrovascular malformations
Conway et al.[9] (2001)	USA	Retrospective	25	30 (at the time of death)	IA diagnosis based on autopsy reports	0	N/A
Rosser et al.[4] (2005)	USA	Retrospective	353 (316)	7.3 (at the diagnosis of vasculopathy)	MRI/MRA. MRI as a routine screening	1	7
Schievink et al.[6] (2005)	USA	Retrospective	39 (22)	30.4 ± 17.6	MRI. Imaging for clinical indication.	2	N/A
Cairns et al.[5] (2008)	Australia	Retrospective	698 (144)	N/A (< 18 years)	MRI/MRA/DSA. Imaging for clinical indication	0	7
Rea et al.[21] (2009)	Australia	Retrospective	419 (266)	N/A (< 18 years)	MRI/MRA. Imaging for clinical indication	1	17
Kaas et al.[22] (2013)	USA	Retrospective	181 (80)	12.2 ± 0.4	MRI/MRA/DSA. Imaging for clinical indication.	0	12
Ghosh et al.[23] (2013)	USA	Retrospective	398 (312)	11.7 ± 7.3 (at the diagnosis of vasculopathy)	MRI/MRA. Imaging for clinical indication.	0	15
Bekiesińska-Figatowska et al.[24] (2013)	Poland	Retrospective	(37)	6.6	MRA. Imaging for clinical indication	0	1
Kim et al.[10] (2016)	USA	Retrospective	(47)	38.3	MRA/CTA/DSA. Imaging for clinical indication	5	N/A

SAH, subarachnoid hemorrhage; ICH, intracerebral hemorrhage, MRI, magnetic resonance imaging; MRA, magnetic resonance angiography; DSA, digital subtraction angiography; CTA, computed tomography angiography

**Table 4:** NF1 patients with intracranial aneurysms.

Author	Country	Age	Intracranial aneurysm	Presenting symptom	Sex
Patil et al.[25] (2015)	India	22	N/A	SAH	M
Conforti et al.[26] (2014)	Italy	22	ICA (fusiform)	Tolosa-Hunt syndrome	M
González-Tortosa et al.[27] (2011)	Spain	23	ICA	Diploic haematoma	F
You et al.[28] (2011)	Korea	17	MCA (saccular), extracranial ICA	–	M
Ellis et al.[29] (2011)	Canada	9	MCA (saccular)	ICH	F
Oderich et al.[7] (2007)	USA	Case 1: 50	ICA bilaterally	Asymptomatic	F
		Case 2: 20	ICA	Asymptomatic	F
		Case 3: 43	MCA, extracranial aneurysms	Asymptomatic	F
		Case 4: 74	ICA bilaterally, basilar artery, extracranial aneurysms	Asymptomatic	M
		Case 5: 43	ICA, MCA, extracranial aneurysms	SAH	F
Baldauf et al.[30] (2005)	Germany	34	ACA (saccular)	SAH	F
Mitsui et al.[31] (2001)	Japan	49	Basilar (fusiform)	Lateral medullary syndrome	M
Zhao and Han[32] (1998)	China	55	ICA (saccular)	SAH	F

SAH, subarachnoid haemorrhage; ICH, intracerebral haemorrhage, MCA, middle cerebral artery; ICA, internal carotid artery; ACA, anterior cerebral artery

Our results indicate that NF1 is not associated with elevated incidence of subarachnoid haemorrhage. However, our data does not make it possible to draw strong conclusions of the impact of NF1 on unruptured IA prevalence. The incidence of NF1 derived from the Finnish hospital registers is 1 per 2000 live births.[16] Due to increased mortality associated with NF1, the overall prevalence of NF1 is considerably less than the birth incidence. NF1 patients have a shortened lifespan: their mean age at death is 52 years in Finland.[16] Pöyhönen et al. have estimated the prevalence of NF1 in different age groups in Northern Finnish population.[33] The observed prevalence varied by age, diminishing from the peak prevalence of about 1/3000 in the age group 10–19 years to 1/10000 in the age group 60–69, with the overall prevalence of 1/4436. Evans et al. reported a similar prevalence estimate of 1/4560.[34] Consequently, without any association between NF1 and IAs, one IA patient with NF1 would be expected–and was found–in the Kuopio IA Database.

Almost all unruptured IA cases are incidental findings in neuroimaging for other reasons and few are large enough to cause neurological symptoms by compression.[19] Only one unruptured IA, detected at autopsy, was observed in the NF1 cohort, even though many of the NF1 patients undergo MRI neuroimaging to rule out intracranial tumours. On the other hand, cranial MRI scans of NF1 patients are often obtained far earlier than IAs would be usually diagnosable. Furthermore, the patients of the NF1 register were not subject to a routine autopsy. There were two confirmed aneurysmal SAH cases among the 1410 NF1 patients as reconstructed from the national registry data of diagnoses; two per 1410 is well in line with the lifetime risk of aSAH in the Finnish population, with an estimated annual incidence of 9 per 100000.[35] In addition, the occurrence of aSAH was not different between the NF1 patients and their matched controls. Consequently, aSAH does not seem to be overrepresented among NF1 patients either.

*NF1* is a tumour suppressor gene whose protein product neurofibromin interacts with the RAS signalling by facilitating the inactivation of the pathway.[3] Since neurofibromin is expressed in the endothelium, localized loss of heterozygosity or haploinsufficiency of *NF1* could lead to excessive intimal thickening of the arterial wall.[36] Stenotic lesions of NF1 patients can be caused by intimal proliferation and fibromuscular dysplasia[37], but although unruptured IA walls often resemble intimal thickening, the initiation of aneurysm formation is characterized by loss of smooth muscle cells and elastic laminas[38]. NF1 is reported to associate with hypertension, an established risk factor for the IA disease.[19,39]

Previous data on possible association between NF1 and IA are based on case reports and small patient series[6,7,10,24,26–32], and have also been contested (Tables 3 and 4).[9] In a retrospective administrative database study from the USA, there were 28 (0,17%) SAH cases among 16918 NF1 patients over 18 years, which is within the range of SAH incidence in general population as well as our NF1 cohort.[11]

Our study has several strengths. Kuopio IA Database reliably reflects the Eastern Finnish IA disease, and the initial screening of IA patients for possible NF1 was conducted with a large array of diagnoses. Furthermore, the 19644 first-degree relatives of the IA patients were reviewed using the national registry data for NF1 as well. Similarly, the NF1 database includes all diagnosed NF1 patients in Finland and their diagnoses have been carefully verified from individual patient records. The data quality of the Finnish national health registers has been shown to be very good.[40]

This study has some limitations. It is theoretically possible that there are more than one NF1 patients among the 4543 IA patients in Kuopio IA Database. The database contains all IA patients admitted to KUH since 1980. The NIH criteria for the diagnosis of NF1 were established in 1987[2] and even after that there may have been NF1 patients who remained undiagnosed. Three IA patients had relatives with NF1 and considering the high penetrance of NF1, subclinical presentation of the disorder cannot be definitely excluded. NF1 cohort was not screened angiographically for the purpose of this study to rule out intracranial vascular malformations and consequently unruptured intracranial aneurysms may have remained unnoticed. We were unable to analyze the number of angiographical screenings the NF1 patients underwent based on our register data. The medical records of the patients of the NF1 register were not analyzed to quantify the number of angiographical screenings these patients underwent. These factors may cause a bias and strong conclusions on the impact of NF1 to unruptured IA incidence cannot be drawn without additional studies. On the other hand, we were able to satisfactorily confirm or exclude aneurysms in all NF1 patients with SAH.

## Conclusions

In conclusion, on the basis of two large Finnish population based cohorts, 4543 IA patients and their 19644 first-degree relatives, as well as the nationwide population-based cohort of 1410 NF1 patients and their 14030 matched controls, there is now evidence that NF1 does not predispose to subarachnoid haemorrhage. No evidence for an association between NF1 and unruptured IAs was found. However, further studies are required to confirm that there is no association between NF1 and unruptured IAs.
